# Elevated serum B-cell activator factor levels predict rapid progressive interstitial lung disease in anti-melanoma differentiation associated protein 5 antibody positive dermatomyositis

**DOI:** 10.1186/s13023-024-03153-6

**Published:** 2024-04-19

**Authors:** Yumeng Shi, Hanxiao You, Chang Liu, Yulu Qiu, Chengyin Lv, Yujing Zhu, Lingxiao Xu, Fang Wang, Miaojia Zhang, Wenfeng Tan

**Affiliations:** 1 Department of Rheumatology, The First Affiliated Hospital of Nanjing Medical University, No. 300, Guangzhou Road, Gulou District, Nanjing, 210029 China; 2 Department of Cardiology, The First Affiliated Hospital of Nanjing Medical University, Nanjing, Jiangsu China

**Keywords:** Biomarker, Dermatomyositis, Melanoma differentiation-associated protein 5 antibody, Rapidly progressive interstitial lung disease, Serum B cell activating factor

## Abstract

**Background:**

Rapid progressive interstitial lung disease (RP-ILD) is the leading cause of anti-melanoma differentiation associated protein 5 antibody positive dermatomyositis (anti-MDA5^+^DM) related death. Elevated serum B-cell activating factor (BAFF) levels have been implicated in connective tissue diseases associated ILD. Here, we evaluate whether BAFF could be a prognostic biomarker for predicting RP-ILD in anti-MDA5^+^DM patients.

**Methods:**

Serums were collected from 39 patients with anti-MDA5^+^DM (20 with RP-ILD and 19 with non-RP-ILD), 20 antisynthase syndrome (ASS) patients and 20 healthy controls (HC). BAFF concentration was measured by an enzyme-linked immunosorbent assay.

**Results:**

Serum BAFF level was higher in anti-MDA5^+^DM patients than those in ASS patients and HC (3882.32 ± 1880.09 *vs.* 2540.89 ± 1403.04 and 2486.28 ± 767.97 pg/mL, *p* = 0.0056 and 0.0038, respectively). Within anti-MDA5^+^DM groups, RP-ILD patients exhibited higher BAFF concentration than non-RP-ILD group (4549.78 ± 1839.97 *vs.* 3297.28 ± 1794.69 pg/mL, *p* = 0.04). The BAFF concentration was positively correlated with levels of C-reactive protein (CRP), dehydrogenase (LDH) and cytokeratin (CK) in anti-MDA5^+^DM patients (*r* = 0.350, *p* = 0.035; *r* = 0.393, *p* = 0.016; *r* = 0.518, *p* = 0.001; respectively). The best cut-off value of BAFF concentration was 2971.5 pg/mL by ROC curve (AUC area = 0.690, *p* = 0.045) and BAFF > 2971.5 pg/mL was an independent risk factor for RP-ILD using multivariate analysis (OR = 9.389, 95% CI = 1.609—54.769; *p* = 0.013).

**Conclusions:**

Serum BAFF could be a useful prognostic biomarker for early detecting RP-ILD risk in anti-MDA5^+^DM patients.

## Introduction

Anti-melanoma differentiation-associated gene 5 antibody positive (anti-MDA5^+^) dermatomyositis (DM) is a specific subtype of DM and is also known as clinically amyopathic dermatomyositis (CADM). Anti-MDA5^+^DM is characterized with subtle or no muscle involvement, but it is associated with a high incidence of interstitial lung disease (ILD). Importantly, approximately 30% of anti-MDA5^+^DM related ILD will develop life-threaten rapidly progressive interstitial lung disease (RP-ILD) [1–5]. Anti-MDA5^+^DM associated RP-ILD is often resistant to glucocorticoids and immunosuppressive therapy. Despite aggressive management, the mortality rate of RP-ILD patients is still as high as 50% to 70%. Therefore, it is imperative to find novel biomarkers for predicting RP-ILD prognosis and to seek new therapeutic targets for preventing progression.

B cell activating factor (BAFF), also known as B lymphocyte stimulator (BLyS), is produced by various immune cells, including monocytes, macrophages, dendritic cells, and T cells [6]. BAFF binds to receptors on the surface of B cells and is crucial for B-cell differentiation, maturation and survival [7]. BAFF transgenic mice could develop the features of systemic lupus erythematosus (SLE) and Sjögren syndrome (SS) [8]. Elevated levels of BAFF have been observed in various autoimmune diseases, including ANCA-associated renal vasculitis, SLE, SS, systemic sclerosis (SSc) and idiopathic inflammatory myopathy (IIM) [7, 9, 10]. Consequently, targeting BAFF signaling has been considered as a promising therapeutic strategy for these diseases.

Recent studies further suggested that serum BAFF levels are associated with connective tissue disease (CTD) associated ILD. Serum levels of BAFF were significantly higher in CTD-ILD patients compared to healthy subjects, and it was inversely correlated with pulmonary function [11, 12]. Overexpression of BAFF was also found in alveolar macrophages and lymphocytes in CTD-ILD patients [11]. SSc patients with elevated serum BAFF levels had decreased vital capacity more frequently [13]. Similarly, DM patients with elevated serum BAFF levels had ILD more frequently [14].

Despite the previously studies have observed the elevated serum levels of BAFF in DM patients, to the best of our knowledge, the relationship between serum BAFF levels and RP-ILD development in anti-MDA5^+^DM has never been reported. In the current study, we investigated the serum BAFF levels in patients with anti-MDA5^+^DM and explored the clinical correlation between serum BAFF level and ILD severity and progression.

## Methods

### Study population

Patients enrolled in this study include 39 anti-MDA5^+^DM patients (27 women and 12 men; mean age, 53.94 ± 13.46 years). Antisynthase syndrome (ASS) is another subtype of idiopathic inflammatory myopathies that is strongly associated with ILD [15], 20 ASS patients (14 women and 6 men; mean age, 54.7 ± 11.87 years), and 20 age and sex matched healthy controls (HC) (11 women and 9 men; mean age, 48.95 ± 11.71 years) was enrolled in the current study as control. The diagnosis of myositis in all patients met the European NeuroMusclar Center (ENMC) criteria or Sontheimer criteria. The study protocol was approved by the First Affiliated Hospital of Nanjing Medical University Committee on Ethics (ID: 2020-SR-265). Informed consent was obtained from each study participant.

Patients were enrolled during their initial visit, at which time blood samples were collected. Additionally, a complete history and physical examination were conducted, accompanied by various laboratory tests. To monitor the progression of interstitial lung disease, follow-up assessments were carried out. Notably, some patients had already been diagnosed and had initiated treatment prior to their initial visit our hospital. Information regarding their medication was recorded.

### Diagnosis of RP-ILD and non-RP-ILD

Anti-MDA5^+^DM patients and ASS patients are divided into RP-ILD and non-RP-ILD (including ILD and non-ILD) subgroups according to the presence of any of the following four conditions within one month of the onset of respiratory symptoms: 1) acute and progressive worsening of dyspnea requiring hospitalization or supplementary oxygen; 2) lung function including forced vital capacity (FVC) decreases by more than 10%, or diffusion capacity for carbon monoxide of the Lung (DLCO) falls over 15% with the decreased FVC; 3) high resolution CT (HRCT) of chest demonstrates that the extent of interstitial abnormalities increased more than 20%; 4) arterial blood gas analysis suggests respiratory failure or the oxygen partial pressure reduction is greater than 10 mmHg. In the anti-MDA5^+^DM group, 20 RP-ILD and 19 non-RP-ILD patients (12 with ILD and 7 with non-ILD) were included. In the ASS group, 6 patients had RP-ILD and 14 patients with non-RP-ILD (12 with ILD and 2 with non-ILD).

### Measurement of BAFF

The blood samples were centrifuged at 3000 rpm for 10 min, then the collected serum samples were stored at -80℃. Serum BAFF concentration (pg/mL) was measured using an enzyme-linked immunosorbent assay (ELISA) test according to the manufacturer’s instructions (BOSTER, Wuhan, China).

### Statistical analysis

Before the analysis, the hypothesis test was carried out by using the normal probability graph to observe whether the test value obeys the normal distribution. The normal distribution measurement data were expressed as mean ± standard deviation (SD), whereas the skewed distribution measurement data were expressed as median (range). The clinical characteristics and lab data of the participants were compared with t test or analysis of variance (ANOVA) for continuous variables and chi-square tests for categorical variables. Univariate and multivariate logistic regression modeling was performed to analyze the relationship between serum BAFF concentration and the risk of RP-ILD as well as other clinical and laboratory parameters. Receiver-operating characteristic (ROC) curve was used to identify the optimal cutoff value of BAFF. Parameters in univariate logistic analysis based on statistical trend with *p* value less than 0.1 were included in the subsequent multivariate analysis. The *p* value below 0.05 was considered as statistically significant. Statistically analyzed and graph drawn using IBM SPSS Statistics 23.0 and GraphPad Prism 8, respectively.

## Results

### Participants characteristics

The clinical characteristics of the participants are shown in Table 1. There was no significant difference in age and gender between the Anti-MDA5^+^DM patients and ASS patients. Prior to enrollment, some patients had already received glucocorticoids or immunosuppressive therapy. Importantly, the types of treatments administered did not differ significantly between the anti-MDA5^+^DM and ASS groups Some patients have already been treated with glucocorticoid or immunosuppressive therapy before enrollment, and there was no significant difference of administered treatments between the anti-MDA5^+^DM patients and ASS patients. Consistent with previous reports, both anti-MDA5^+^DM and ASS patients were prone to concurrent ILD, and the incidence of RP-ILD was higher in the MDA5 group, although no statistical difference was found (51.28% vs. 30%,* p* = 0.120). The proportion of myasthenia, Gottron’s sign, Heliotrope rash, V sign and periungual erythema was higher in anti-MDA5^+^DM groups than those in ASS patients (35.89% *vs.* 5%, *p* = 0.01 for myasthenia; 58.9% *vs.* 20%, *p* = 0.004 for Gottron’s sign; 28.2% *vs.* 5%, *p* = 0.044 for Heliotrope rash; 38.46% *vs.* 5%, *p* = 0.006 for V sign; 25.64% *vs.* 0%, *p* = 0.012 for periungual erythema, respectively). Moreover, serum ferritin (SF) was markedly increased in anti-MDA5^+^DM group (*n* = 39) as compared with those in ASS group (*n* = 20) (916.94 ± 771.48 *vs.* 258.20 ± 156.77 ng/mL, *p* = 0.001) (Table 1). When anti-MDA5^+^DM patients were divided into RP-ILD (*n* = 20) and non-RP-ILD groups (*n* = 19), patients with RP-ILD exhibited significantly higher serum CRP levels than those non-RP-ILD patients [8.81 (2.93–31.88) *vs.* 3.96 (2.17–7.99) mg/L, *p* = 0.021] (Table 2) at baseline. All-cause mortality rate of all anti-MDA5^+^DM patients was 25.64%. All deaths occurred in RP-ILD group due to respiratory failure. There were no statistically significant differences in other clinical manifestations between anti-MDA5^+^DM patients with RP-ILD and with non-RP-ILD.

**Table 1:** Clinical manifestations and laboratory features

Parameters	NC	ASS	MDA5^+^	*p*-value
Case number	20	20	39	
Gender, female, no. (%)	11(55%)	14(70%)	27(69.23%)	0.704
Age, mean ± SD, years	48.95 ± 11.71	54.70 ± 11.87	53.94 ± 13.46	0.834
Medical history, months		10.90 ± 16.35	3.00 ± 2.75	0.051
Medication				
- glucocorticoids, no. (%)		8 (40%)	18 (46.15%)	0.652
- immunosuppressants, no. (%)		7(35%)	13 (33.33%)	0.898
Interstitial lung disease, no. (%)				0.120
non-ILD		2 (10%)	7 (17.95%)	
ILD		12 (60%)	12 (30.77%)	
RP-ILD		6 (30%)	20 (51.28%)	
Myasthenia, no. (%)		1 (5%)	14 (35.89%)	0.010
Gottron’s sign, no. (%)		4 (20%)	23 (58.90%)	0.004
Heliotrope rash, no. (%)		1 (5%)	11 (28.20%)	0.044
V sign, no. (%)		1 (5%)	15 (38.46%)	0.006
Shawl sign, no. (%)		1 (5%)	10 (25.64%)	0.079
Periungual erythema, no. (%)		0 (0%)	10 (25.64%)	0.012
Skin ulcers, no. (%)		0 (0%)	7 (17.95%)	0.083
Mechanic’s hands, no. (%)		4 (20%)	13 (33.33%)	0.284
Arthritis, no. (%)		1 (5%)	10 (25.64%)	0.079
CK, median (range), IU/L		64.00 (39.75–92.50)	47.00 (29.00–105.00)	0.659
LDH, median (range), U/L		268.50 (210.25–367.00)	319.00 (276.00–418.00)	0.070
ESR, median (range), mm/H		36.00 (7.00–45.00)	36.00 (25.00–48.75)	0.761
SF, mean ± SD, ng/mL		258.20 ± 156.77	916.94 ± 771.48	0.001
CRP, median (range), mg/L		6.30 (1.62–27.82)	4.94 (2.30–11.40)	0.692
BAFF, mean ± SD, pg/mL	2486.28 ± 767.97	2540.89 ± 1403.04	3882.32 ± 1880.09	0.001

Data are presented as mean ± SD or median (range) or case number (percentage); Student’s t-test, Pearson’s Chi square test and analysis of variance (ANOVA) were used to analysis. All of the clinical and laboratory parameters were obtained at the first evaluation

*Anti-MDA5*^+^*DM* anti-melanoma differentiation-associated protein 5 antibody positive dermatomyositis, *RP-ILD* rapidly progressive interstitial lung disease, *CK* cytokeratin, *CRP* C-reactive protein, *LDH* dehydrogenase, *ESR* erythrocyte sedimentation rate, *SF* serum ferritin, *BAFF* B-cell activating factor

**Table 2:** Clinical manifestations and laboratory features

Parameters	non-RP-ILD	RP-ILD	*p*-value
Case number	19	20	
Gender, female, no. (%)	13 (68.42%)	14 (70%)	0.915
Age, mean ± SD, years	51.05 ± 16.05	56.70 ± 10.10	0.194
Medical history, months	3.73 ± 2.90	2.35 ± 2.07	0.103
Medication			
- glucocorticoids, no. (%)	9 (47.37%)	9 (45%)	0.882
- immunosuppressants, no. (%)	7 (36.84%)	6 (30%)	0.651
Myasthenia, no. (%)	5 (26.31%)	9 (45%)	0.224
Gottron’s sign, no. (%)	13 (68.42%)	10 (50%)	0.242
Heliotrope rash, no. (%)	5 (26.31%)	6 (30%)	0.798
V sign, no. (%)	9 (47.36%)	6 (30%)	0.265
Shawl sign, no. (%)	6 (31.57%)	4 (20%)	0.408
Periungual erythema, no. (%)	5 (26.31%)	5 (25%)	0.925
Skin ulcers, no. (%)	5 (26.31%)	2 (10%)	0.184
Mechanic’s hands, no. (%)	6 (27.27%)	7 (35%)	0.821
Arthritis, no. (%)	6 (21.21%)	4 (20%)	0.408
CK, median (range), IU/L	47.00 (35.00–122.00)	51.00 (25.50–98.25)	0.561
LDH, median (range), U/L	317.00 (253.00–418.00)	319.00 (277.25–423.00)	0.853
ESR, median (range), mm/H	33.50 (22.00–46.50)	41.00 (21.00–54.00)	0.348
SF, median (range), ng/mL	586.10 (453.25–876.70)	916.40 (641.75–1121.93)	0.692
CRP, median (range), mg/L	3.96 (2.17–7.99)	8.81 (2.93–31.88)	0.021
BAFF, mean ± SD, pg/mL	3297.28 ± 1794.69	4549.78 ± 1839.97	0.040

Data are presented as mean ± SD or median (range) or case number (percentage). Student’s t-test, Pearson’s Chi square test and analysis of variance (ANOVA) were used to analysis. All of the clinical and laboratory parameters were obtained at the first evaluation

*Anti-MDA5*^+^*DM* anti-melanoma differentiation-associated protein 5 antibody positive dermatomyositis, *RP-ILD* rapidly progressive interstitial lung disease, *CK* cytokeratin, *LDH* dehydrogenase, *ESR* erythrocyte sedimentation rate, *SF* serum ferritin, *CRP* C-reactive protein, *BAFF* B-cell activating factor

### Elevated serum BAFF levels in anti-MDA5^+^DM

**Fig. 1:**
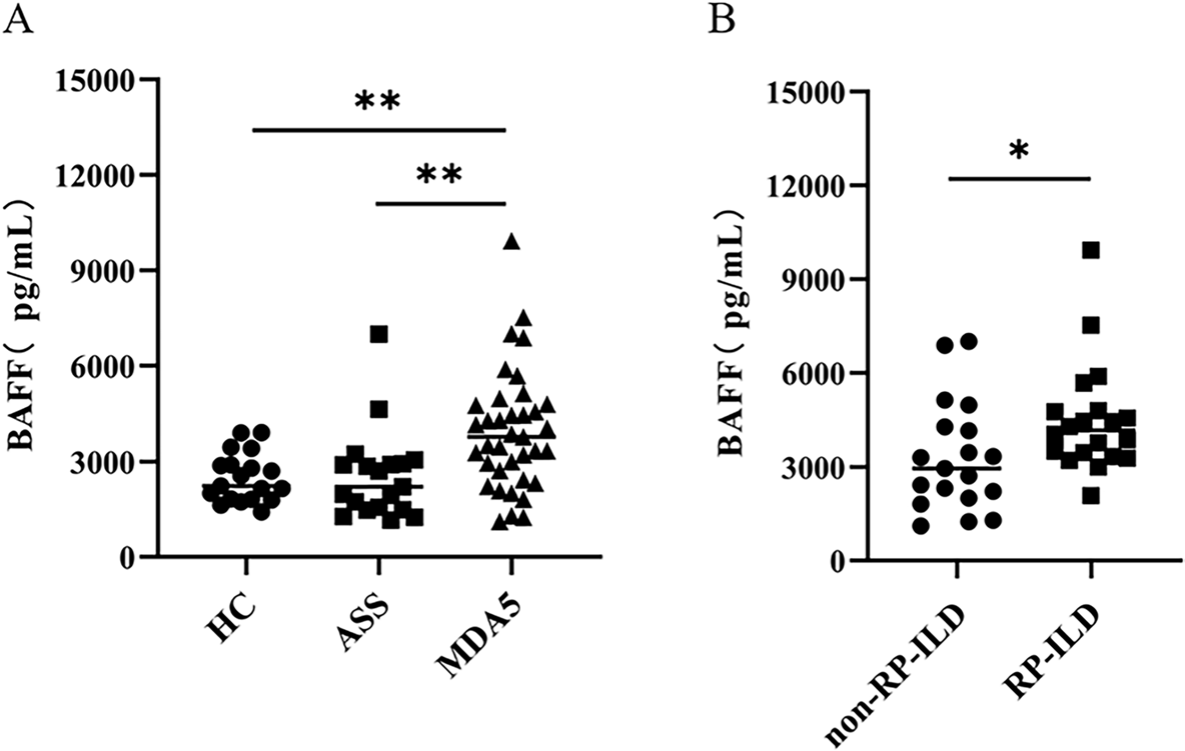
Serum BAFF concentration in HC, ASS and anti-MDA5^+^DM patients. **A** Serum BAFF levels in HC (*n* = 20), ASS (*n* = 20) and anti-MDA5^+^DM patients (*n* = 39); **B** Serum BAFF concentrations in anti-MDA5^+^DM patients with (*n* = 20) and without RP-ILD (*n* = 19); **p < 0.01; *p < 0.05

Serum levels of BAFF was significantly higher in patients with anti-MDA5^+^DM than those in patients with ASS (3882.32 ± 1880.09 *vs.* 2540.89 ± 1403.04, *p* = 0.0056) or HC (3882.32 ± 1880.09 *vs.* 2486.28 ± 767.97 pg/mL, *p* = 0.003) (Fig. 1 and Table 1). No significant difference in BAFF levels was found between ASS patients and HC. When patients were divided into RP-ILD and non-RP-ILD groups, the RP-ILD patients had the significantly higher BAFF levels as compared to non-RP-ILD patients (4549.78 ± 1839.97 *vs.* 3297.28 ± 1794.69 pg/mL, *p* = 0.04) (Fig. 1 and Table 2).

### Clinical correlation of serum BAFF levels in anti-MDA5^+^DM

**Fig. 2:**
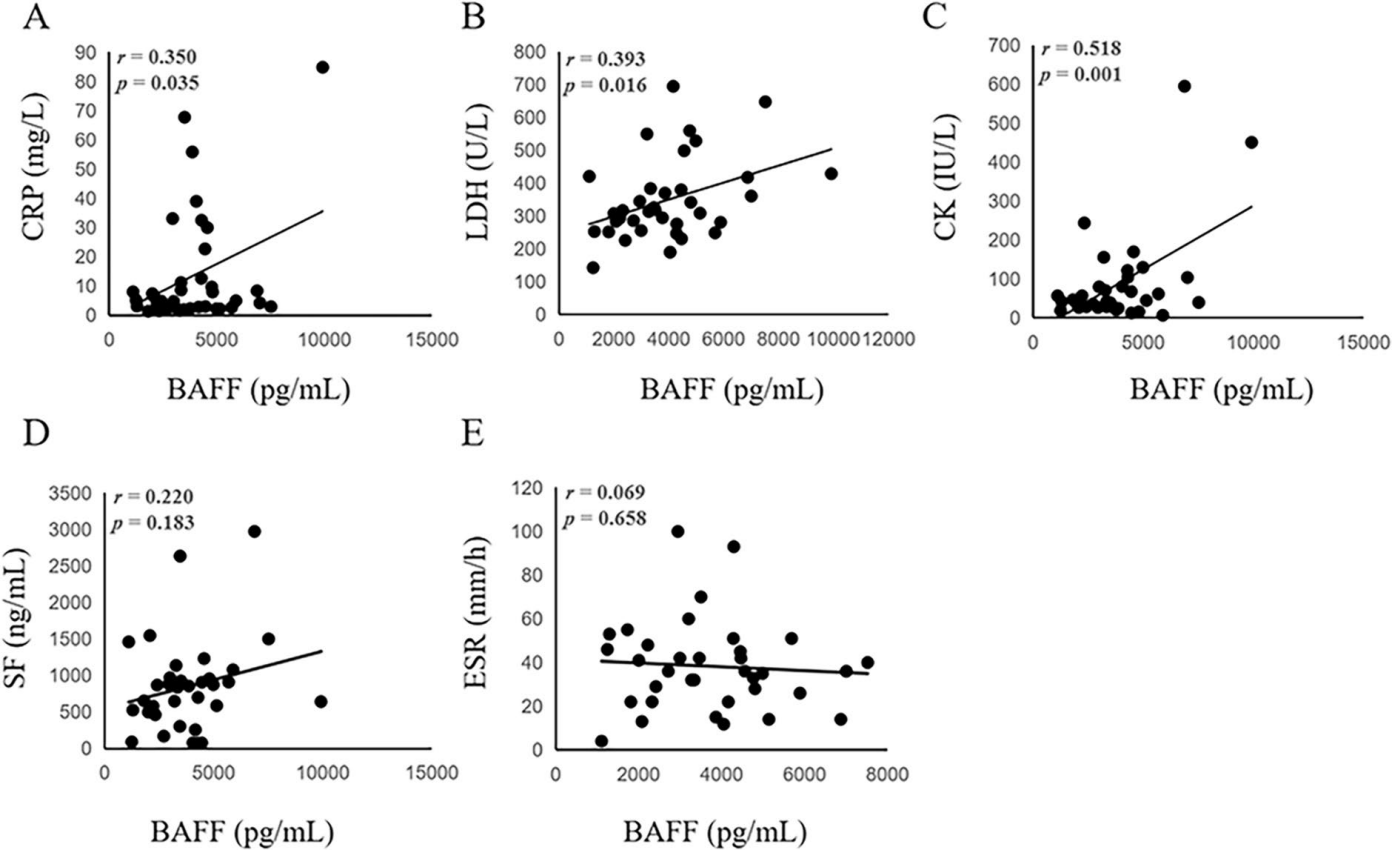
The correlation of serum BAFF and disease activity in anti-MDA5^+^DM. Correlation of serum BAFF levels with serum levels of CRP (**A**), LDH (**B**), CK (**C**), SF (**D**), ESR (**E**) in patients with anti-MDA5^+^DM at the first evaluation. Serum BAFF levels were determined by a specific ELISA

To explore the characteristics of patients with high serum BAFF levels, we evaluated the correlation between serum BAFF levels and clinical parameters. The results showed that BAFF levels were correlated positively with the serum levels of CK, LDH and CRP in anti-MDA5^+^DM patients (*r* = 0.518, *p* = 0.001 for CK;* r* = 0.393, *p* = 0.016 for LDH; *r* = 0.350, *p* = 0.035 for CRP; respectively) (Fig. 2A-C). However, neither serum levels of serum ferritin (SF) nor erythrocyte sedimentation rate (ESR) level was significantly correlated with serum BAFF levels (*p* = 0.183 and *p* = 0.658, respectively) (Fig. 2D-E).

### Independent predictability of BAFF to RP-ILD in anti-MDA5^+^DM

**Fig. 3:**
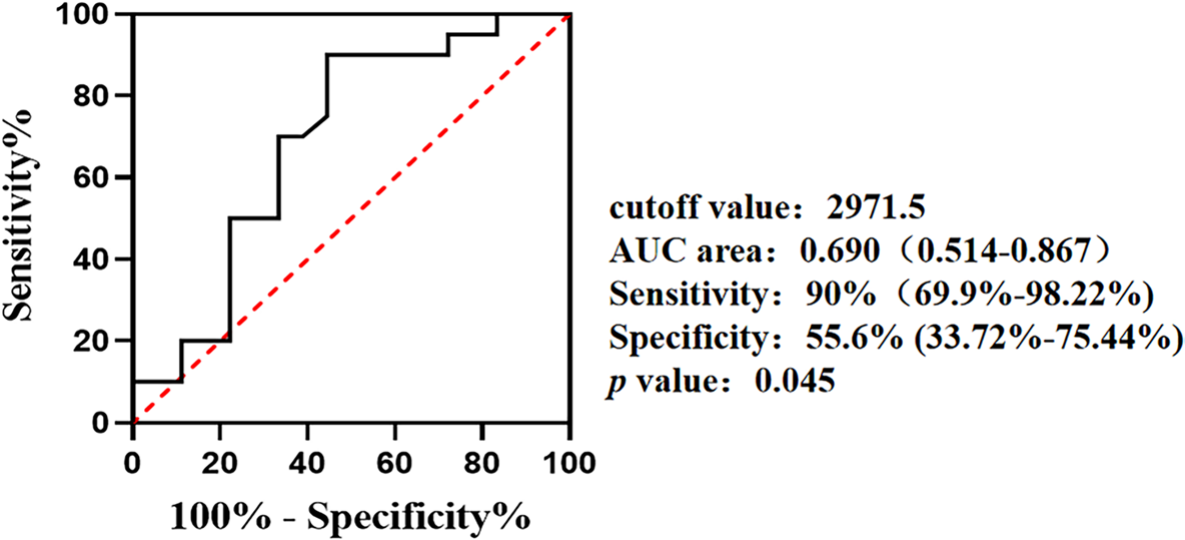
ROC curve of serum BAFF concentration in anti-MDA5^+^DM patients. ROC curve shows the area under the curve (AUC) of serum BAFF concentration in anti-MDA5^+^DM patients based on patients whether development of RP-ILD

In order to predict the occurrence of RP-ILD in anti-MDA5^+^DM, ROC curve was used to determine the cut-off value of serum BAFF concentration. The data showed that 2971.5 pg/mL has the highest diagnostic efficiency to distinguish the patients with or without RP-ILD (AUC area = 0.690, 95% CI = 0.514–0.867, sensitivity 90% and specificity 55.6%; *p* = 0.045) (Fig. 3). In order to further clarify the application efficiency of the above cut-off value in anti-MDA5^+^DM patients with RP-ILD, we next performed the logistic regression analysis. In the logistic regression model, the upper limit of the normal range of the healthy population for the continuous variables (CK, LDH, ESR, CRP, SF) was used as the cutoff value for the binary classification. After continuous variables were transformed into dichotomies, the results of univariate analysis showed that CRP > 8 mg/L and BAFF > 2971.5 pg/mL were candidate risk factors for anti-MDA5^+^DM with RP-ILD. When they were included into the multivariate equation, BAFF > 2971.5 pg/mL was an independent risk factor for RP-ILD in anti-MDA5^+^DM patients (OR = 9.389, 95% CI = 1.609–54.769; *p* = 0.013) (Table 3).

**Table 3:** Logistic analysis of rapidly progressive interstitial lung disease influenced by characteristics of dermatomyositis patients

RP-ILD	Unadjusted		Adjusted	
	OR (95% CI)	*p*-value	OR (95% CI)	*p*-value
Gender, Female	1.077 (0.276–4.197)	0.915		
Age	1.034 (0.988–1.078)	0.198		
Myasthenia	2.291 (0.595–8.825)	0.228		
Gottron’s sign	0.462 (0.125–1.703)	0.246		
Heliotrope rash	1.350 (0.379–4.811)	0.643		
V sign	0.690 (0.213–2.237)	0.536		
shawl signs	0.675 (0.174–2.614)	0.570		
Periungual erythema	1.333 (0.341–5.208)	0.679		
arthritis	0.674 (0.174–2.614)	0.570		
mechanic hands	1.185 (0.382–3.675)	0.769		
skin ulcers	0.467(0.083–2.627)	0.387		
CK, > 200 U/L	0.593 (0.088–4.009)	0.592		
LDH, > 227 IU/L	2.235 (0.186–26.908)	0.526		
ESR, > 21 mm/h	1.133(0.198–6.486)	0.888		
CRP, > 8 mg/L	3.750 (0.917–15.342)	0.066	2.337 (0.487–11.228)	0.289
SF, > 336.2 ng/ml	1.373 (0.462–4.073)	0.568		
BAFF, > 2971.5 pg/ml	11.250 (1.991–63.560)	0.006	9.389 (1.609–54.769)	0.013

Binary logistical regression analysis was used in regression equation. CRP > 8 mg/L and BAFF > 2971.5 pg/mL were put into the multivariate regression analysis

*Anti-MDA5*^+^*DM* anti-melanoma differentiation-associated protein 5 antibody positive dermatomyositis, *RP-ILD* rapidly progressive interstitial lung disease, *CK* cytokeratin, *LDH* lactate dehydrogenase, *ESR* erythrocyte sedimentation rate, *CRP* C-reactive protein, *SF* serum ferritin, *BAFF* B-cell activating factor

## Discussion

In the present study, we measured serum BAFF level and explored its clinical implication in patients with anti-MDA5^+^DM. We found that serum BAFF levels were significantly increased in anti-MDA5^+^DM patients compared with those in ASS patients and HC. Moreover, serum BAFF level was associated with disease severity of ILD, and BAFF levels > 2971.5 pg/mL was an independent risk factor for RP-ILD in anti-MDA5^+^DM patient. These findings indicated that BAFF participates in the pathological process of ILD and might serve as a biomarker for RP-ILD risk in anti-MDA5^+^DM patients.

Previous studies have revealed that serum BAFF level was elevated in DM, and associated with the prevalence of ILD [16, 17]. The major finding of current study is that serum BAFF level was significant positive correlation with RP-ILD in anti-MDA5^+^DM. These findings have at least 2 important clinical implications.

First, our present results suggest that serum BAFF had certain predictive value for RP-ILD and poor prognosis in patients with anti-MDA5^+^DM. As a life-threatening complication of anti-MDA5^+^DM, early recognition and early treatment of RP-ILD is the key to improve prognosis. Although several serum risk factors, including anti-MDA5 antibody titer, CRP, LDH, and SF [18–20], are thought to be associated with the development of ILD in MDA5^+^ patients, however, they are not a good predictor of the occurrence of RP-ILD. In current study, with a cut-off value of 2971.5 pg/ml of serum BAFF can help distinguish RP-ILD patients from anti-MDA5^+^DM patients (Fig. 3). Multivariate regression further suggested that BAFF was an independent risk factor for RP-ILD in anti-MDA5^+^DM.

Second, our finding of an association of RP-ILD with elevated serum imply BAFF-blocking therapy could be an attractive novel treatment for anti-MDA5^+^DM patients**,** especially patients with a tendency toward RP-ILD**.** Elevated CD19^+^ B cells have been found in anti-MDA5^+^DM patients compared with ASS, and are associated with poor outcomes [21]. As a salvage therapy strategy, Rituximab is usually selected for the treatment of RP-ILD with a resistance to conventional therapy or with a life-threatening condition [22]. However, the uncertain efficacy and high risk of infection limits the widespread adoption of B-cell depletion in clinical practice.

BAFF plays an important role in the activation and homeostasis of B cell. The increased serum BAFF level is significantly correlated with disease-specific antibodies level in some autoimmune diseases, such as anti-SSA in SS, anti-dsDNA in SLE, anti-histone in SSc [23–26] and anti-Jo1 in IIM [27–29]. It is thought that BAFF may contribute to the development of RP-ILD in anti-MDA5^+^DM by promoting the survival and activation of autoreactive B cells, and then enhance the production of a variety of autoantibodies, including anti-MDA5 autoantibodies. Besides, BAFF contributes to progression of ILD by impairing apoptosis of naive B cells via BAFF receptor [30]. Additionally, BAFF also promotes pulmonary interstitial fibrosis by acting as a potent inducer of TIMP-1, α-SMA, CCL2, and IL-6 [31]. Therefore, more research is needed to determine the long-term safety and efficacy of BAFF inhibition in the treatment of anti-MDA5^+^DM associated RP-ILD.

We also found that BAFF levels were correlated positively with the serum level of CRP, CK and LDH in anti-MDA5^+^DM patients. We previously reported elevated serum CRP and LDH levels represented the high inflammation condition in anti-MDA5^+^DM patients and linked to RP-ILD and poor outcomes [32]. Consistent with these findings, the current data indicate that excessive BAFF level is involved in the inflammatory response or autoimmune reaction in anti-MDA5^+^DM patients. Combined serum BAFF levels with other serum markers including CRP and LDH may reflect severity of lung injury and can help early identify RP-ILD patients in anti-MDA5^+^DM patients.

This study had several limitations. Due to the low incidence of MDA5, we included a limited number of cases in this study. Besides, lung function tests were not performed in all patients and there was a lack of correlation between BAFF levels and FVC or DLCO values, which have been reported as risk factors for RP-ILD combined with anti-MDA5^+^DM [33]. In addition, a minority of patients had already been treated with glucocorticoid or immunosuppressive therapy at the time of enrollment. The potential influence of these therapy on BAFF levels presents a confounding factor that did not be analyzed in this study. Moreover, we did not obtain the dynamic change of BAFF level with treatment response and the number of effector B cells at the matched time point. These results are need to further validation in a large and prospective cohort.

## Conclusions

The current study revealed that elevated serum BAFF levels are associated with an increased risk of developing RP-ILD. Therefore, combining serum BAFF levels with imaging and clinical features may aid the early detection of high-risk RP-ILD patients. Furthermore, BAFF may be a potential target for preventing RP-ILD in anti-MDA5^+^DM patients. Further research is needed to fully understand the role of BAFF in RP-ILD development, and explore the potential benefits and risks of BAFF inhibition in anti-MDA5^+^DM patients.

## Data Availability

All data generated or analyzed during this study are included in this published article.
